# Role of social capital in the association between ageism and depressive symptoms among older adults in South Korea

**DOI:** 10.1093/geroni/igaf122.2211

**Published:** 2025-12-31

**Authors:** Bokyoung Choi, Jihwan Lee, Jihee Choi, Eunhee Choi, Chang-O Kim, Soong-nang Jang

**Affiliations:** Chung-Ang University, Seoul, Republic of Korea; Chung-Ang University, Seoul, Republic of Korea; Institute for Community Care and Health Equity, Seoul, Republic of Korea; Institute for Community Care and Health Equity, Seoul, Republic of Korea; Dolbom Clinic, Seoul, Republic of Korea; Chung-Ang University, Seoul, Republic of Korea

## Abstract

**Background:**

Ageism is a known risk factor for mental health in older adults, but the role of social capital in this relationship remains unclear. This study examines how bridging and bonding social capital moderate the association between perceived ageism and depressive symptoms.

**Methods:**

Using data from the 2023 National Survey of Older Koreans, we analyzed the association between perceived ageism (interpersonal and institutional) and depressive symptoms (SGDS-15) among adults aged 65 and older. Perceived ageism was categorized into interpersonal ageism and institutional ageism based on the context in which discrimination occurred, including public institution, hospital, workplace, and home. We also examined the moderating effects of bridging social capital (e.g., participation in senior welfare centers) and bonding social capital (e.g., membership in social or hobby clubs). Prevalence ratios (PR) and 95% confidence intervals (CI) were calculated.

**Results:**

Among participants, 47.5% reported interpersonal ageism, while 27.5% reported institutional ageism. Interpersonal ageism was associated with 2.92 times higher odds of depressive symptoms, and institutional ageism with 1.98 times higher odds. However, the relationship between ageism and depressive symptoms varies depending on the type of social capital. Bridging social capital attenuated this association to non-significance, whereas bonding social capital exacerbated it, amplifying the adverse impact of ageism on depressive symptoms.

**Conclusion:**

Bridging social capital may act as a protective factor against the negative effects of ageism, whereas bonding social capital may reinforce them. These findings underscore the need for expanding older adults’ social networks beyond close-knit groups to mitigate the psychological impact of ageism.

